# Tissue microarray is suitable for scientific biomarkers studies in endometrial cancer

**DOI:** 10.1007/s00428-017-2289-6

**Published:** 2018-02-09

**Authors:** Nicole C. M. Visser, Anneke A. M. van der Wurff, Johanna M. A. Pijnenborg, Leon F. A. G. Massuger, Johan Bulten, Iris D. Nagtegaal

**Affiliations:** 1 Department of Pathology, Radboud university medical center, P.O. Box 9101, 6500 HB Nijmegen, the Netherlands; 2 Department of Pathology, Elisabeth-TweeSteden Hospital, Tilburg, the Netherlands; 3 Department of Obstetrics and Gynecology, Radboud university medical center, Nijmegen, the Netherlands

**Keywords:** Endometrial cancer, Endometrial sampling, Tissue microarray, Biomarkers, Immunohistochemistry

## Abstract

The aim of this study was to define the concordance between tissue microarrays (TMAs) of different sizes and whole slide for 15 different antibodies in endometrial cancer and study the use of TMAs in preoperative endometrial samples. Cores of preoperative and hysterectomy specimens of 14 endometrial cancer and three atypical hyperplasia cases were collected in TMA blocks. Two 0.6-mm and two 2.0-mm cores were used from each sample. Different antibodies were tested in TMAs and compared with results of whole slides of hysterectomy. Tested antibodies were as follows: ER, PR, p53, Ki-67, MLH1, PMS2, MSH2, MSH6, ARID1A, stathmin, IMP3, L1CAM, PTEN, β-catenin, and p16. Seventeen cases with four cores per paraffin block (both 0.6 and 2.0 mm in duplicate) and 15 different antibodies resulted in a total of 1020 cores for both preoperative and hysterectomy specimen. Overall, 2.0-mm cores were more assessable for evaluation than 0.6-mm cores (96.0 versus 79.5%, *p* < 0.01). For most antibodies, a substantial to good agreement between hysterectomy TMA and whole slide was present, with lowest agreement for p16 and stathmin and perfect agreement for mismatch repair proteins. Preoperative TMAs showed for most antibodies moderate to perfect agreement with hysterectomy TMAs. In conclusion, 2.0-mm cores are the preferred size for immunohistochemical studies in endometrial cancer. For all tested antibodies, TMAs are a good alternative for whole slide analysis in scientific studies with large patient cohorts, even in preoperative endometrial samples. However, caution is required for interpretation of TMA results of p16 and stathmin.

## Introduction

In endometrial cancer, biomarkers may add information for risk stratification for tailored treatment strategies [1]. Many biomarkers for individualized treatment in endometrial cancer were discovered recently [1]. To validate these biomarkers, we need large-scale studies with examination of many samples which requires a large amount of tissue and laboratory consumables that induces high research costs. Tissue microarrays (TMAs) are an efficient way to examine a large number of cases on a single slide. In 1998 Kononen et al. described the use of TMAs for high-throughput, molecular characterization of a large panel of tumor specimens [2].

Many studies have compared TMAs to whole sections [3–6]. However, different core sizes for TMAs can be used and limited information is available about comparison of different core sizes [7, 8]. Furthermore, primary treatment is based on preoperative diagnosis, with a discordance between preoperative and postoperative diagnosis in 15–40% of the cases [9–12]. This can result in both over- and undertreatment. Biomarkers might minimize this discordance. However, most studies have studied new biomarkers in hysterectomy specimens [3]. Yet, frequently, endometrial sampling reveals only scant material [13, 14]. This makes the use of TMAs in preoperative endometrial samples a challenge.

The aim of the present study is to define the concordance between TMAs of different sizes and whole slide for 15 different antibodies and study the use of TMAs in preoperative endometrial samples.

## Material and methods

### Tissue samples

Cases were retrieved from the pathology archive of the Radboud university medical center, Nijmegen, the Netherlands. Seventeen cases, 14 with primary endometrial cancer and 3 with atypical hyperplasia of the endometrium who have had preoperative endometrial sampling and hysterectomy, were included in this study. We selected representative cases (*n* = 14) of different types of endometrial cancer, including four endometrioid endometrial cancer (EEC) grade 1, four EEC grade 2, two EEC grade 3, two serous carcinoma (USC), and two clear-cell carcinoma (CCC). From each case, a representative block with tumor of both the preoperative and the hysterectomy specimen was collected. We selected only cases with sufficient tumor tissue in the paraffin block. The cases were reviewed by a specialized gynecopathologist (JB).

### Tissue microarray construction

From each case, four representative areas with tumor or hyperplasia were encircled on the hematoxylin and eosin (H&E) slide of both the preoperative and hysterectomy specimen. TMAs were constructed using the TMA grand master (3DHISTECH, Budapest, Hungary). From each corresponding paraffin block, two 0.6-mm and two 2.0-mm cores were made. This resulted in four TMA blocks: one TMA block containing 17 duplicate 0.6-mm cores of the preoperative specimen, one TMA block containing 17 duplicate 2.0-mm cores of the preoperative specimen, one TMA block containing 17 duplicate 0.6-mm cores of the hysterectomy specimen, and one TMA block containing 17 duplicate 2.0-mm cores of the hysterectomy specimen. Seventeen cases with four cores per paraffin block (both 0.6 and 2.0 mm in duplicate) and 15 different antibodies result in a total of 1020 stained cores for both preoperative and hysterectomy specimen.

### Immunohistochemistry and scoring

Immunohistochemistry was performed on 4-μm sections of the four TMA blocks and the corresponding whole slides of the hysterectomy, using 15 different antibodies (Table 1). In short, antigen retrieval was performed and endogenous peroxidase blocked with hydrogen peroxide. Slides were incubated with the primary antibody for 1 h at room temperature. Subsequently, they were incubated with PowerVision+ Poly-HRP and visualized with PowerVision DAB substrate solution (Leica Biosystems, Buffalo Grove, IL, USA). Finally, the slides were counterstained with hematoxylin, dehydrated, and mounted.

**Table 1:** Antibodies used in this study

Antibody	Staining pattern	Clone	Antigen retrieval	Dilution	Source	Interobserver variability (kappa)
ER	Nuclear	SP1	EDTA pH 9	1:80	Immunologic	0.959
PR	Nuclear	PgR636	EDTA pH 9	1:500	Dako	0.961
MLH1	Nuclear	G168-15	EDTA pH 9	1:40	BD	0.894
PMS2	Nuclear	A16-4	EDTA pH 9	1:100	BD	0.800
MSH2	Nuclear	GB12	EDTA pH 9	1:20	Calbiochem	0.848
MSH6	Nuclear	EPR3945	EDTA pH 9	1:1000	Abcam	0.763
PTEN	Nuclear and cytoplasmic	6H2.1	EDTA pH 9	1:100	Dako	0.724
IMP3	Cytoplasmic	69.1	EDTA pH 9	1:50	Dako	0.912
β-Catenin	Membranous and cytoplasmic	14/β-catenin	EDTA pH 9	1:200	BD	0.911
L1CAM	Membranous	14.10	EDTA pH 9	1:100	BioLegend	0.921
Ki-67	Nuclear	MIB-1	Citrate pH 6.0	1:40	Dako	0.998
ARID1A	Nuclear	HPA005456	Citrate pH 6.0	1:100	Sigma	0.796
Stathmin	Cytoplasmic	3351	Citrate pH 6.0	1:50	Cell Signaling	0.824
P53	Nuclear	DO-7	Citrate pH 6.7	1:200	Immunologic	0.901
P16	Nuclear and cytoplasmic	MX007	HIER pH 8.0	1:60	Immunologic	0.779

All TMA cores and whole slides were scored twice, by two independent investigators (NCMV in combination with JB, IDN, or AAMW). In case of discrepancies, the slides were discussed together and a consensus score was made. We have used a semiquantitative scoring index to evaluate the immunohistochemical staining, with exception of the mismatch repair (MMR) proteins, ARID1A, and Ki-67. The scoring index (0–9) was obtained by the product of intensity, graded from 0 (no staining), 1 (weak staining), 2 (moderate staining), to 3 (strong staining), and area of tumor with this staining intensity, graded from 0 (no positive tumor cells), 1 (1–10% positive tumor cells), 2 (11–50% positive tumor cells), to 3 (> 50% positive tumor cells). We designated cutoff values of the scorings index (0–9) for positivity/high expression as follows: ER, PR, IMP3, L1CAM, and p16 scoring index ≥ 4, PTEN ≥ 2, β-catenin ≥ 6, and stathmin ≥ 7. For p53, aberrant staining was defined as a scorings index ≥ 5 or complete negative staining. For ARID1A and MMR (MLH1, PMS2, MSH2 and MSH6) tumors were considered aberrant if tumor cells showed complete absence of nuclear staining, with positive internal control (stromal cells). Tumors were considered positive for ARID1A and MMR if there was any positive tumor cell with nuclear staining, regardless of intensity. Cases with negative tumor cells and no internal control were scored as indeterminate. For Ki-67, the percentage of positive tumor cells was determined. Results of duplicate cores of each tumor were combined to give a tumor score. In case the two scores differed, the mean of the two scores was used as the overall tumor score. If one core was not assessable, the overall tumor score was that of the remaining assessable core. Cores were considered assessable if there was enough tumor tissue for evaluation of the immunohistochemical staining. Cores were considered not assessable in case of < 10% tumor cells in the core (“sampling error,” e.g., only stroma) or when < 10% of the tissue was present in the core (“absent core”).

### Statistical analysis

Difference in assessability between 0.6- and 2.0-mm cores was calculated with chi-squared test. The agreement between the immunohistochemical score on TMA and whole slide, and between preoperative and hysterectomy TMA, was determined by calculating Cohen’s kappa. Agreement was considered poor if *κ* < 0.2, fair if 0.21 < *κ* < 0.4, moderate if 0.41 < *κ* < 0.6, substantial if 0.61 < *κ* < 0.8 and almost perfect if 0.81 < *κ* < 1.00. Since Ki-67 score was not dichotomized, the difference in Ki-67 score between whole slide and TMA was calculated with the related-samples Wilcoxon signed rank test. The difference in ratio between false positive and false negative cases was also calculated with the related-samples Wilcoxon signed rank test. A *p* value of < 0.05 was considered to indicate statistical significance. Statistical analysis was performed with SPSS version 22 (SPSS IBM, New York, NY, USA).

## Results

### Assessability

Of the 1020 tumor cores with core size 0.6 mm, 811 were assessable (79.5%), whereas 979 of the tumor cores with core size 2.0 mm were assessable (96.0%) (*p* < 0.01). The difference in assessability was more prominent in the hysterectomy TMAs than the preoperative TMAs; however, both show a significant difference (*p* < 0.01). Of the hysterectomy TMAs 71.2% of the 0.6-mm cores and 98.6% of the 2.0-mm cores was assessable, compared to respectively 87.8 and 93.3% of the preoperative TMAs. The most common cause for a not assessable core was tumor loss during process (10%). In only 1% of the cases, there was sampling error with less than 10% tumor cells.

### Interobserver variability

There was a substantial to almost perfect agreement between the TMA score of the different investigators. Kappa values varied between 0.72 for PTEN to 0.998 for Ki-67 (Table 1).

### Heterogeneity

**Fig. 1:**
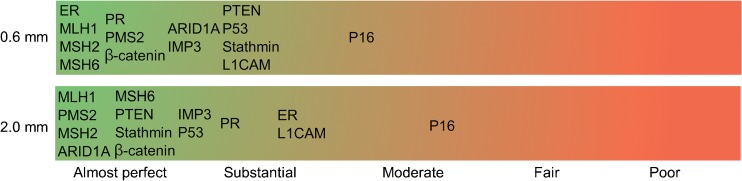
Strength of agreement between two cores of the same size (kappa statistics). Green indicates almost perfect agreement, red indicates poor agreement

Overall, there was a good agreement between scores of cores of the same size. The agreement varied per antibody, between moderate for p16 to almost perfect for MLH1, PMS2, MSH2, MSH6, β-catenin, IMP3, and ARID1A (Fig. 1). For some antibodies, the agreement was better with larger cores compared to 0.6-mm cores (PTEN, ARID1A, Stathmin, p53), whereas others show better agreement with smaller cores (ER, PR, MSH6).

### Preoperative versus hysterectomy TMAs

**Fig. 2:**
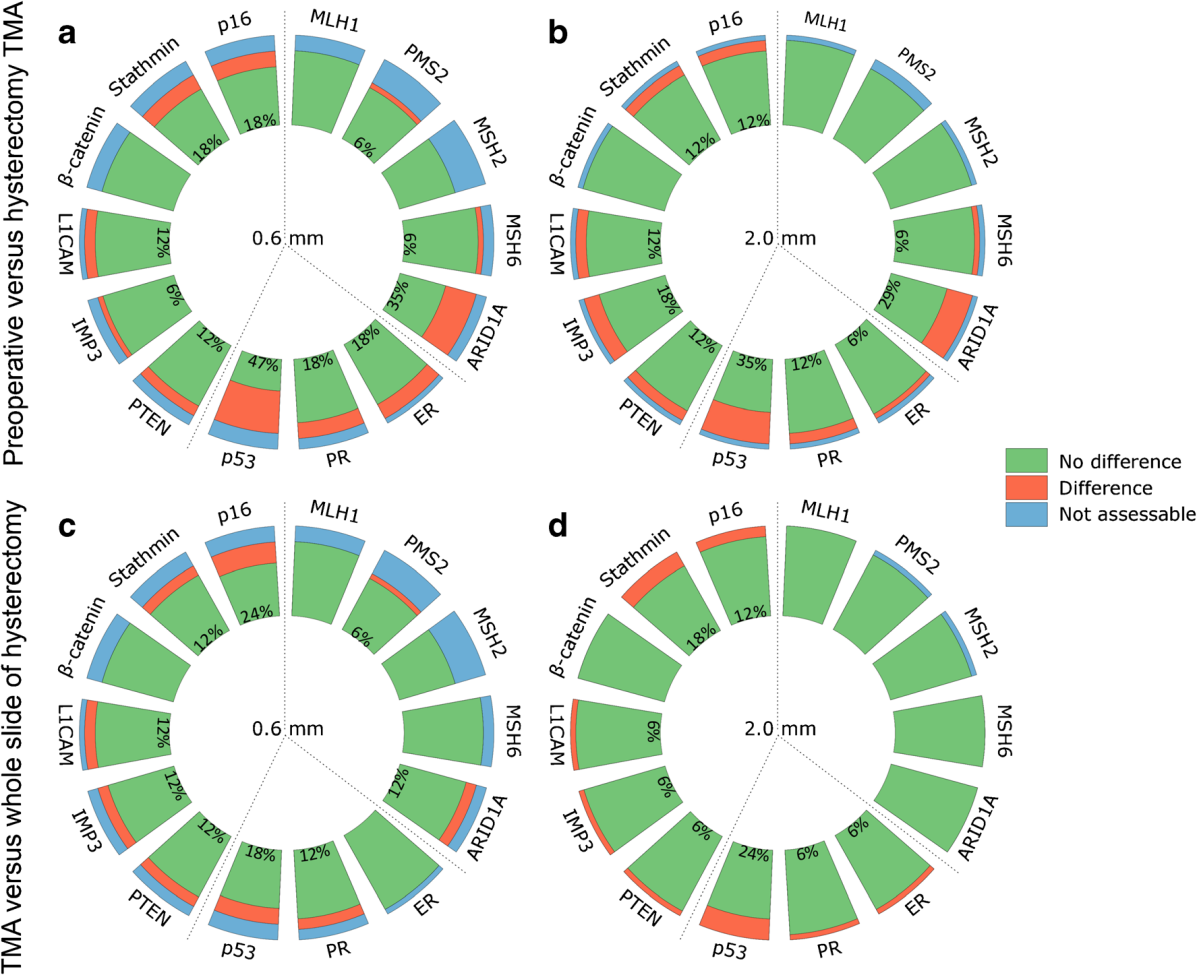
Preoperative versus hysterectomy TMA for 0.6 mm (**a**) and 2.0 mm (**b**) and TMA versus whole slide of hysterectomy for 0.6-mm (**c**) and 2.0-mm cores (**d**). Green indicates no difference, red indicates difference, and blue indicates not assessable cores. Percentages represent the percentage of cores with a difference between respespectively preoperative and hysterectomy TMA score and between TMA and whole slide of hysterectomy

The correlation between preoperative TMAs and hysterectomy TMAs differs per antibody and varied from almost perfect agreement for MLH1, PMS2, MSH2, β-catenin, and ER to poor agreement for ARID1A, p53, and stathmin (Fig. 2a, b). There were more false positive than false negative cases on preoperative TMAs (*p* = 0.04). Overall, 9% of the cases were false positive and 5% were false negative on preoperative TMA. False positive rates per antibody varied from 0 to 36% and false negative rates from 0 to 21%. ARID1A and P53 staining showed the highest false positive rate (respectively 33 and 36%). For Ki-67, the score was significantly higher on preoperative TMA compared to hysterectomy TMA, both for 2.0- and 0.6-mm cores (*p* = 0.001). The median difference between the Ki-67 score on preoperative TMA and hysterectomy TMA was larger in 2.0-mm compared to 0.6-mm cores (respectively 36 and 11, *p* < 0.05) (not shown in figure).

### TMA versus whole slide of hysterectomy

**Fig. 3:**
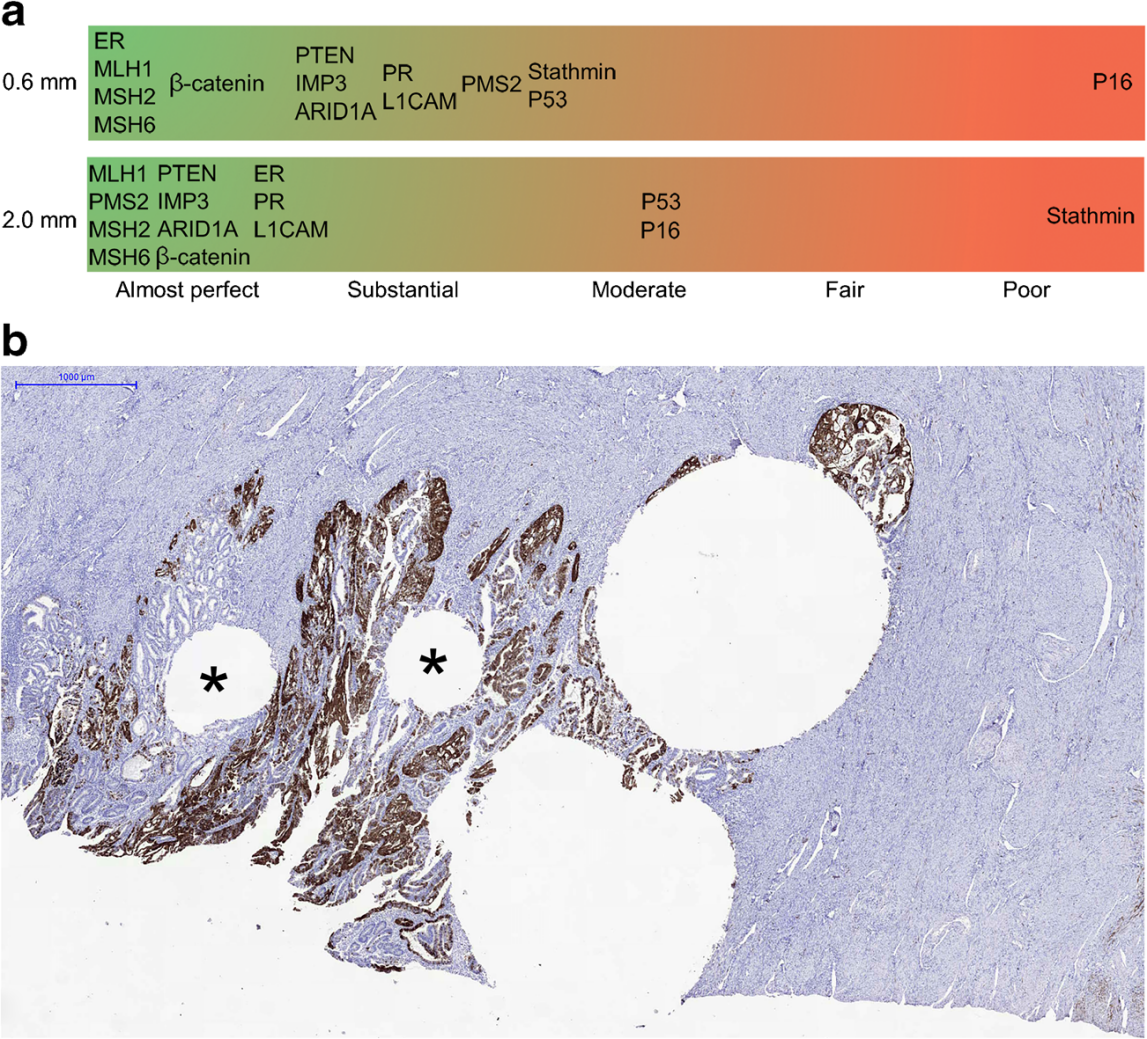
TMA versus whole slide of hysterectomy. **a** Strength of agreement between TMA and whole slide of hysterectomy (kappa statistics). **b** Case with discrepancy between whole slide and 0.6 mm TMA for p16 (two 0.6-mm core holes marked with an asterisk). Whole slide showing positive staining (scorings index 6), concordant positive 2.0-mm cores (combined scorings index 6) and discrepant negative 0.6-mm cores (combined scorings index 3)

For most antibodies, there was a substantial to good agreement between hysterectomy TMA and whole slide, with exception of stathmin, p16, and p53 (Figs. 2c, d and 3a). Stathmin and p16 staining show a moderate to poor agreement between TMA and whole slide of hysterectomy, depending on the core size. For p16, all but one were scored positive on whole slide. Four of the 14 assessable cases were scored negative for p16 on 0.6-mm TMA cores (Fig. 3b). For stathmin, all cases were scored negative on 2.0-mm TMA cores. Three of these 17 cases were scored positive on whole slide. Most discrepancies for p53 were seen for cases with complete negative, aberrant staining on TMA and positive staining on whole slide (*n* = 4).

**Fig. 4:**
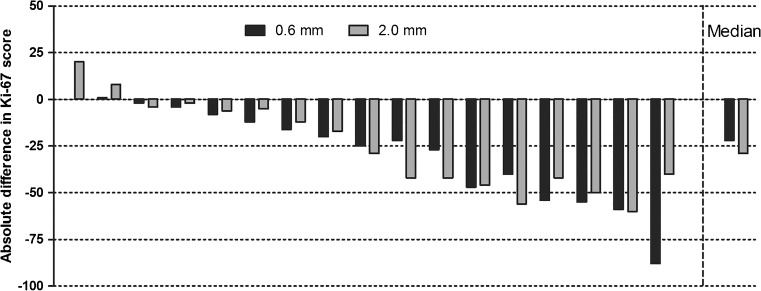
Absolute difference in Ki-67 score between TMA and whole slide (ref.) per case. Each bar represents a case, with in black the 0.6-mm cores and in gray 2.0-mm cores. In the most right column, the median difference in Ki-67 for all cases, separated by core diameter

For Ki-67, the score was significantly lower on TMA than on whole slide, for both 2.0- and 0.6-mm cores (respectively *p* < 0.01) (Fig. 4). The median difference between the Ki-67 score on TMA and whole slide was 29 and 22 for 2.0- and 0.6-mm cores, respectively, which is not significantly different.

## Discussion

Overall, 2.0-mm cores were more assessable for evaluation than 0.6 mm cores (96.0 versus 79.5%, *p* < 0.01). For most antibodies, a substantial to good agreement between hysterectomy TMA and whole slide was present. Preoperative TMAs showed for most antibodies moderate to perfect agreement with hysterectomy TMAs.

TMAs are an efficient method to test new biomarkers in a research setting, since it saves time, costs, and tissue [15]. This last issue is also one of the concerns. Tumors can be heterogeneous and low density or diffuse tumors might be difficult to sample. In endometrial cancer, many promising biomarkers have been reported the last years [16–20]. Most of these biomarkers have been studied in hysterectomy specimens, whereas especially preoperative biomarkers might be clinically useful to choose the best surgical treatment. Previous studies have reported a significant correlation for stathmin, L1CAM, ER, and PR expression between curettage and hysterectomy specimen [18, 21, 22]. However, although there is a correlation, expression pattern is not completely overlapping, with a discordance rate of 10% for L1CAM to 33% for stathmin. These percentages are in line with the results of the present study. In general, we reported more false positive than false negative cases on preoperative TMAs (respectively 9 and 5%). Although based on small numbers, this difference was most pronounced for p53 and ARID1A. Studies that describe discrepancies between preoperative and hysterectomy specimen show different results about the false positive and false negative rates [18, 21–23]. Differences in staining between preoperative and hysterectomy specimen could be explained by differences in fixation or the representativeness of the tissue. With endometrial sampling, only the superficial part of the tumor is examined, whereas in a hysterectomy specimen also the invasive front is examined. Moreover, tumor heterogeneity can be a challenge when using TMAs for biomarker studies. Distinction between true intratumor heterogeneity and irregular staining can be challenging. In case of alternate groups of tumor cells with complete negative and positive staining on the whole slide, we considered intratumor heterogeneity as the cause of discrepancy. In case of a gradual decrease in staining intensity on whole slide, we considered irregular staining the most likely cause of the discrepancy. In our study, discrepancies between TMA and hysterectomy could be attributed to both causes.

Although p16 is a heterogeneous staining, it still showed a moderate agreement between the two cores. To decrease the effect of heterogeneity on TMA score, investigators have the dilemma to use more or larger cores or perform the staining on whole slides. Especially for antibodies such as p16 and stathmin, additional cores or staining on whole slide should be considered to lower the chance of false negative cases.

There is no consensus on the optimal number of cores. Kyndi et al. reported that a single core seems to be sufficient, whereas Neves-Silva et al. reported that two cores per case is the optimal number with regard to tissue loss and agreement with whole slide [24, 25]. The same goes for core size. Most studies report that smaller cores, in case assessable, are as representative as larger cores. However, the relation between core loss after immunohistochemical staining and core size is less clear. Neves-Silva et al. shows more loss in 1.0-mm cores than 2.0-mm cores, whereas Fonseca et al. reported that core loss increases with increasing core size from 3 to 7% with cores of respectively 1.0 and 3.0 mm [8, 24]. In the present study, we reported more core loss when using 0.6-mm cores compared to 2.0-mm cores. An explanation might be that larger cores have better adhesion to the surrounding paraffin of the donor block because of the larger diameter. Moreover, core loss might also be dependent on the type of specimen used. For example, TMAs of large solid tumors might show more assessable cores than a TMA from a small biopsy. Although the amount of preoperative tissue is often scant, cores were lost in only 7 to 12% of the cases. This is in line with literature that report 2–25% core loss [3, 5, 15, 26].

One of the limitations of this study is the small number of cases. However, for most antibodies, there were positive and negative cases. Only for β-catenin, all cases were positive, which was expected, since Shaco-Levy reported beta-catenin positivity in all endometrioid carcinomas and all proliferative endometrium cases [27]. Due to the strong cytoplasmatic staining for β-catenin, evaluation of nuclear staining was not reliable. Therefore, only cytoplasmatic and membranous staining was scored for β-catenin.

Per case, we have created duplicate cores of both 0.6 and 2.0 mm to gain information about the optimal number and size of the TMA cores. We have not stained the whole slide of the preoperative specimen because after punching out the cores for the TMAs in most cases, only a limited amount of tumor was left in the paraffin block. Therefore, we could only compare the hysterectomy TMA to whole slide staining.

The strength of this study is the large number of cores (1020) and antibodies that were studied. To our knowledge, this is the first study that systematically studies the use of TMAs for biomarkers studies in endometrial cancer, with special attention to the use on preoperative tissue. Because of the high discrepancy between preoperative and final diagnosis in endometrial cancer, preoperative risk classification should be improved to prevent over- and undertreatment. New biomarkers might help to improve this risk classification [28]. TMAs might help in discovering and validating new biomarkers, since most of the time, large patient cohorts are required for validation studies.

In conclusion, based on the more frequent loss of cores with smaller core size, 2.0-mm cores are the preferred size for immunohistochemical studies in endometrial cancer. Agreement between TMA and whole slide, and preoperative and hysterectomy TMAs varied per antibody, with lowest agreement for p16 and stathmin and perfect agreement for mismatch repair proteins. For all tested antibodies TMAs are a good alternative for whole slide analysis in scientific studies with large patient cohorts, even in preoperative endometrial samples with clearly less tissue than in a hysterectomy specimen. However, caution is required for interpretation of TMA results of p16 and stathmin.
